# *In vitro* morphology, viability and cytokine secretion of uterine telocyte‐activated mouse peritoneal macrophages

**DOI:** 10.1111/jcmm.12711

**Published:** 2015-10-16

**Authors:** Chi Chi, Xiao‐Juan Jiang, Lei Su, Zong‐Ji Shen, Xiao‐Jun Yang

**Affiliations:** ^1^ Department of Obstetrics and GynecologyThe First Affiliated Hospital of Soochow UniversitySuzhou cityJiangsu provinceChina

**Keywords:** uterine telocytes, macrophage activation, immunoregulation, immune response, cytokines, enzyme, infertility, fertility disorder

## Abstract

Telocytes (TCs), a distinct interstitial cell population, have been identified in the uterus, oviduct and placenta, with multiple proposed potential biological functions. Their unique structure allows them to form intercellular junctions with various immunocytes, both in normal and diseased tissues, suggesting a potential functional relationship with the local immune response. It has been hypothesized that through direct heterocellular junctions or indirect paracrine effects, TCs influence the activity of local immunocytes that are involved in the inflammatory process and in immune‐mediated reproductive abnormalities. However, no reliable cytological evidence for this hypothesis is currently available. In this study, we cultured primary murine uterine TCs and collected TC conditioned media (TCM). Mouse peritoneal macrophages (pMACs) were co‐cultured for 48 hrs with TCM or with DMEM/F12 or lipopolysaccharide (LPS) as negative and positive controls, respectively. Normal uterine TCs with a typical structure and a CD‐34‐positive/vimentin‐positive/c‐kit‐negative immunophenotype were observed during culture. Morphologically, TCM‐treated pMACs displayed an obvious activation/immunoresponse, in contrast to over‐stimulation and cell death after LPS treatment and no sign of activation in the presence of DMEM/F12. Accordingly, a cell counting kit 8 (CCK‐8) assay indicated significant activation of pMACs by TCM and LPS compared to DMEM/F12, thus supporting the marked morphological differences among these groups of cells. Furthermore, within a panel of macrophage‐derived cytokines/enzymes, interleukin‐6 (IL‐6) and inducible nitric oxide synthase were significantly elevated in TCM‐treated pMACs; tumour necrosis factor α, IL1‐R1, and IL‐10 were slightly, but significantly, up‐regulated; and no changes were observed for transforming growth factor‐β1, IL‐1β, IL‐23α and IL‐18. Our results indicate that TCs are not simply innocent bystanders but are rather functional players in the activation of pMACs; they trigger and maintain the immune response, likely through indirect paracrine effects. Thus, we provide preliminary *in vitro* evidence of immunoregulatory and immunosurveillance roles for TCs.

## Introduction

Telocytes (TCs) represent a distinct interstitial cell population that is present in a wide variety of human and mammalian reproductive organs/tissues, including non‐pregnant and pregnant myometrium, oviduct and placenta 1, 2, 3, 4, 5, 6, 7, 8, 9, 10, 11, 12, 13, 14, 15, 16, 17, 18, 19, 20, 21, 22, 23, 24, 25, with multiple proposed potential biological functions. Telocytes have extremely long, thin cytoplasmic extensions called telopodes (Tps) that provide a visible direct structural support for homocellular or heterocellular junctions, potentially contributing to the maintenance of local homeostasis, tissue repair/remodelling and intercellular signalling 26, 27, 28, 29, 30, 31, 32, 33, 34, 35, 36, 37, 38, 39, 40, 41, 42, 43, 44, 45, 46, 47, 48, 49, 50, 51, 52, 53, 54, 55, 56, 57, 58, 59, 60, 61, 62. However, a growing number of studies have described indirect intercellular communication by TCs that involves chemical 34, 35, 63 and paracrine/juxtacrine signalling 5, 34, 35, 36, 37, 38, extracellular vesicles (EVs) 4, 5, 35, 36, 37, 38, 39, 40, 41, 42, 43, 44, 45, 46, 63, 64, 65, 66, sex hormones 3, 5, 18, 35, 67 and/or microRNAs 28, 41, 68, 69, 70. Specifically, EVs and/or exosomes are shed or released from Tps in uterine TCs 4, 5, 43; soluble mediators, such as IL‐6, VEGF and nitric oxide, are secreted from TCs 28, 51, 71; and growth factors, including IL‐6, VEGF, macrophage inflammatory protein 1α (MIP‐1α), MIP‐2 and Monocyte Chemoattractant Protein 1 (MCP‐1), are significantly expressed along with additional cytokines, including IL‐2, IL‐10, IL‐13, and chemokines, such as Growth‐Related Oncogene/Keratinocyte‐derived Chemokine (GRO‐KC), in the secretome of cultured rodent cardiac TCs 71. These paracrine effects might contribute to function‐specific intercellular communication and regulate the activity of neighbouring cells.

Accumulating studies have shown that in both normal and diseased tissues, TCs develop intercellular contacts with various immunocytes, including macrophages, mast cells and lymphocytes 7, 8, 9, 10, 11, 12, 44, 45, 46, 47, 48, 49, 50, 51, 52, 53, 54, 55, 56, 72. Telocytes might modulate the activity of immunocytes through direct intercellular junctional complexes or indirect paracrine communication 45, 46, 47, 48, 49. Telocytes might be active players in local immunoregulation and immunosurveillance, acting as important ‘local data suppliers’ for the immune response 44, 46, 51, 52. It is conceivable that TCs are involved in the pathological processes of multiple autoimmune, chronic inflammatory and fibrotic disorders, and progressive local loss of TCs might contribute to altered intercellular communication or disrupted immune homeostasis 10, 11, 12, 44, 50, 52, 53, 54, 72.

Infertility is the most common disease that affects women of reproductive age. Among the possible causes of infertility, immune‐mediated fertility problems and related diseases, such as endometriosis, pelvic inflammatory disease and salpingitis, are prevalent in the clinic. We were the first to report 11, 12 that TCs were connected to activated immunocytes, including mononuclear cells, mast cells, eosinophils and neutrophils, *via* heterocellular junctions in inflammatory‐affected oviduct tissue from an Spraque‐Dawley (SD) rat model; these data suggested the potential involvement of TCs in local immuno‐inflammatory processes. Through direct heterocellular junctions or indirect paracrine effects, TCs might influence local immunological microenvironments, participate in immunological signal presentation and/or transduction, and contribute to subsequent immune responses and immune‐mediated gynecological diseases or reproductive abnormalities. Nevertheless, no reliable cytological evidence is currently available to support this hypothesis. Herein, we evaluate the *in vitro* paracrine effects of uterine TCs on mouse peritoneal macrophage (pMAC) morphology, viability and cytokine/enzyme production. This study aimed to provide *in vitro* evidence for the immunoregulatory/immunosurveillance roles of uterine TCs.

## Materials and methods

### Culture of uterine telocytes

Animal care, surgery and handling procedures were approved by the University Health Network Animal Care Committee. Adult female BALB/c mice (8–10 weeks old, 20–25 g) were provided by the Laboratory Animal Center of Soochow University. All mice were maintained in a specific pathogen‐free environment with *ad libitum* access to food and water before the experiments. To obtain primary uterine TCs, mice were killed with an overdose of sodium pentobarbital (50 mg/kg; Fuyang Pharmaceutical Factory, Fuyang, China), and uterine tissue was removed and rinsed three times with PBS containing 100 U/ml penicillin and 0.1 mg/ml streptomycin (Sigma‐Aldrich, St. Louis, MO, USA). Uterine samples were then placed in a plastic dish containing sterile PBS and subjected to mechanical grinding (with a particle size of <1 mm^3^); next, tissue fragments were collected in a sterile tube (Corning, New York, USA) and centrifuged at 179 g for 5 min. The supernatants were removed, and the pellet was re‐suspended in DMEM/F12 (Gibco, New York, USA) containing 0.1% collagenase type II (Sigma‐Aldrich). Tissue digestion was performed at 37°C with vigorous shaking at 9 g for 90 min. and gentle agitation using a Pasteur pipette every 15 min. The enzymatic reaction was terminated by the addition of 10% FBS (Gibco). The cells were harvested by centrifugation at 302 g for 10 min., re‐suspended in 5 ml of DMEM/F12 supplemented with 10% FBS and antibiotics, plated in 25 cm^2^ cell culture flasks (Corning), and maintained in a humidified atmosphere containing 5% CO_2_ at 37°C for 90 min. The culture medium was removed, the cells were rinsed twice, and 5 ml of complete medium was added. The medium was changed every 48 hrs, at which point the cells were examined using a microscope (Leica, Heidelberg, Germany).

### Methylene blue staining for viability

Cultured TCs were washed with pre‐warmed phenol red‐free DMEM/F12, fixed and stained in methylene blue solution (0.05 mg/ml; Sigma‐Aldrich) at 37°C for 20 min., and imaged.

### Mitochondrial labelling

MitoTracker Green FM (Beyotime, Shanghai, China) was used to label mitochondria. The cells were incubated in phenol red‐free DMEM with 100 nmol/l MitoTracker Green in a humidified atmosphere with 5% CO_2_ at 37°C for 30 min. Then, the cells were examined and photographed using fluorescence microscopy (450–490 nm excitation, 520 nm barrier filter; Leica).

### Immunofluorescence cytochemistry

Freshly harvested cells were treated with 0.25% Trypsin‐ethylenediaminetetraacetic acid (Gibco) and plated at a low density on coverslips for 48 hrs, followed by fixation in acetone for 2 min., and permeabilization with 0.5% Triton X‐100 for 1 hr. Immunostaining was simultaneously performed with rat anti‐vimentin (1:200; cat. no. ab115189; Abcam, Cambridge, MA, USA) and rabbit anti‐CD34 (1:200; cat. no. ab81289; Abcam). Fixed cells were incubated with the primary antibodies for 90 min. at 37°C and then with goat anti‐rat IgG‐FITC (1:400; cat. no. sc‐2011; Santa Cruz Biotechnology, Santa Cruz, CA, USA) and goat anti‐rabbit IgG‐CY3 (1:400; cat. no. ab97075; Abcam) for 30 min. at 37°C. Finally, the cells were counterstained with DAPI (1:50; cat. no. C1002) and mounted with antifade medium (1:1000; cat. no. p0126; both from Beyotime). Similar procedures were applied for dual antibody labelling with rat anti‐vimentin (1:200; cat. no. ab115189) and rabbit anti‐c‐kit (1:200; cat. no. ab5506; both from Abcam). The stained slides were observed under the aforementioned fluorescence microscope.

### Preparation of telocyte conditioned media

Within 3–4 days of primary cell culture, TCs entered the logarithmic growth phase. At this point, the TCs were plated in 6‐well culture plates (1 × 10^6^ cells/well) containing 2.5 ml of serum‐free DMEM/F12. After a 24 hrs incubation at 37°C, TC conditioned media (TCM) was harvested and stored at −80°C.

### Isolation and co‐culture of peritoneal macrophages

Peritoneal macrophages were isolated from BALB/c mice. Briefly, 2 ml of 3% thioglycollate (Sigma‐Aldrich) was injected into the abdominal cavity to activate pMACs and thus enable the harvest of an optimal yield; after 3 days, the mice were killed with an overdose of sodium pentobarbital *via* a subcutaneous injection. Then, 15 ml of cold sterile DMEM/F12 was injected intraperitoneally, and peritoneal lavage fluids were collected using sterile syringes 10 min. later.

The number of pMACs was counted microscopically using trypan blue dye. Then, pMACs were seeded in 96‐well plates (1 × 10^5^ cells/well) and 12‐well plates (1 × 10^6^ cells/well) for cell counting kit 8 (CCK‐8) assays and ELISAs for cytokines/enzymes, respectively. Briefly, after incubation at 37°C for 4 hrs to allow pMACs to adhere to the surface of the plastic culture plates, the non‐adherent cells were removed. The remaining adherent pMACs (>98%) were cultured with TCM, serum‐free DMEM/F12 (negative control), or serum‐free DMEM/F12 with 0.5 μg/ml lipopolysaccharide (LPS, a classical inflammatory stimulus; positive control) (Sigma‐Aldrich). At 24 and 48 hrs, pMACs from different groups were subjected to morphology and viability assays. Then, culture supernatants were collected by filtration through a microporous film with a 0.22 μm pore size and stored at −80°C for ELISAs for a panel of cytokines/enzymes.

### Cell viability assay

Ten microliters of CCK‐8 solution (Dojindo Laboratories, Tokyo, Japan) was added to each well containing 100 μl of medium. The absorbance at 450 nm of each well was monitored using a micro‐well plate reader (Multiscan MK3; Thermo Labsystems, Waltham, MA, USA) after a 2 hrs incubation at 37°C.

### Measurement of cytokines/enzymes in supernatants

A panel of nine pMAC‐related cytokines/enzymes, including inducible nitric oxide synthase (iNOS), IL‐6, tumour necrosis factor α (TNF‐α), IL1‐R1, IL‐10, transforming growth factor (TGF)‐β1, IL‐1β, IL‐23α and IL‐18, was analysed using commercially available ELISA kits (EIAab, Wuhan, China) according to the manufacturer's instructions.

### Statistical analysis

The data are presented as the mean ± S.D., and the data were analysed by one‐way anova or Student's *t*‐test using SPSS (version 13; SPSS Inc., Chicago, IL, USA), followed by Dunnett's test for comparisons between the DMEM/F12 and test groups. *P* < 0.05 was considered to be statistically significant.

## Results

### *In vitro* TC identification

**Figure 1:**
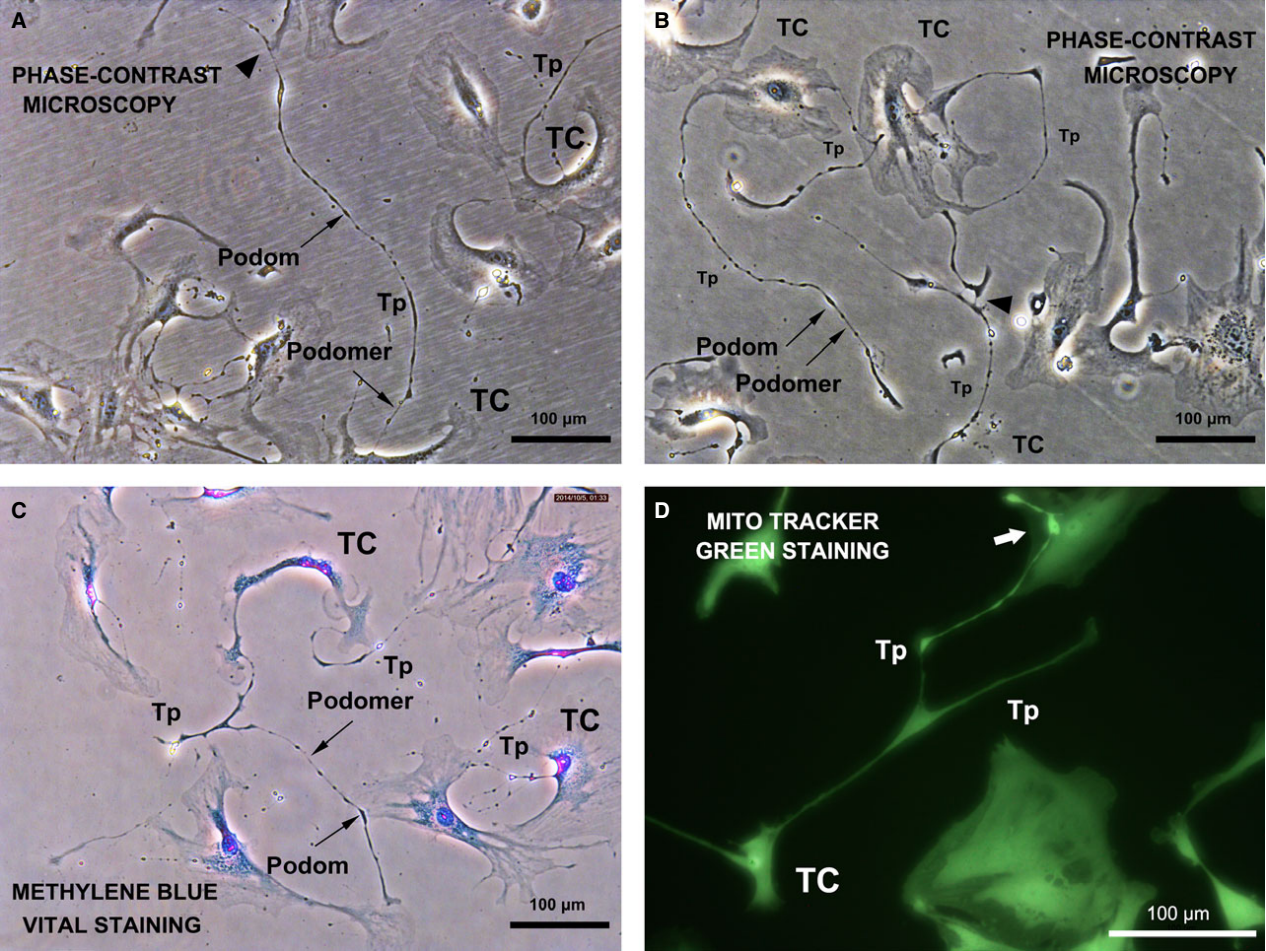
Phase‐contrast microscopy of typical live TCs in primary culture; mouse uterus. TCs have irregularly shaped cell bodies and long characteristic Tps extending from the cell body, with typical alternating podoms (thick segments) and podomers (thin segments) that form a homocellular network (black arrowhead). (**A** and **B**) phase‐contrast microscopy. (**C**) phase‐contrast microscopy with methylene blue staining. (**D**) TCs with long, thin branches (Tps). High‐intensity fluorescence was observed around the intercellular connections between Tps and other cells, indicating active energy metabolism (white arrow). Fluorescence microscopy, MitoTracker green staining.

After 3 or 4 days of primary cell culture, uterine TCs were clearly identified based on morphology. The TCs had small bipolar or multipolar cellular bodies with one or more extremely long, thin, sinuous cellular projections called Tps, which were composed of alternating thin segments (podomers) and thick segments (podoms). Telocytes use their Tps to establish homocellular contacts with adjacent TCs or Tps (Fig. 1A–C). Furthermore, numerous mitochondria were observed (Fig. 1D), indicating active cell metabolism.

### TCs immunodiagnostics

**Figure 2:**
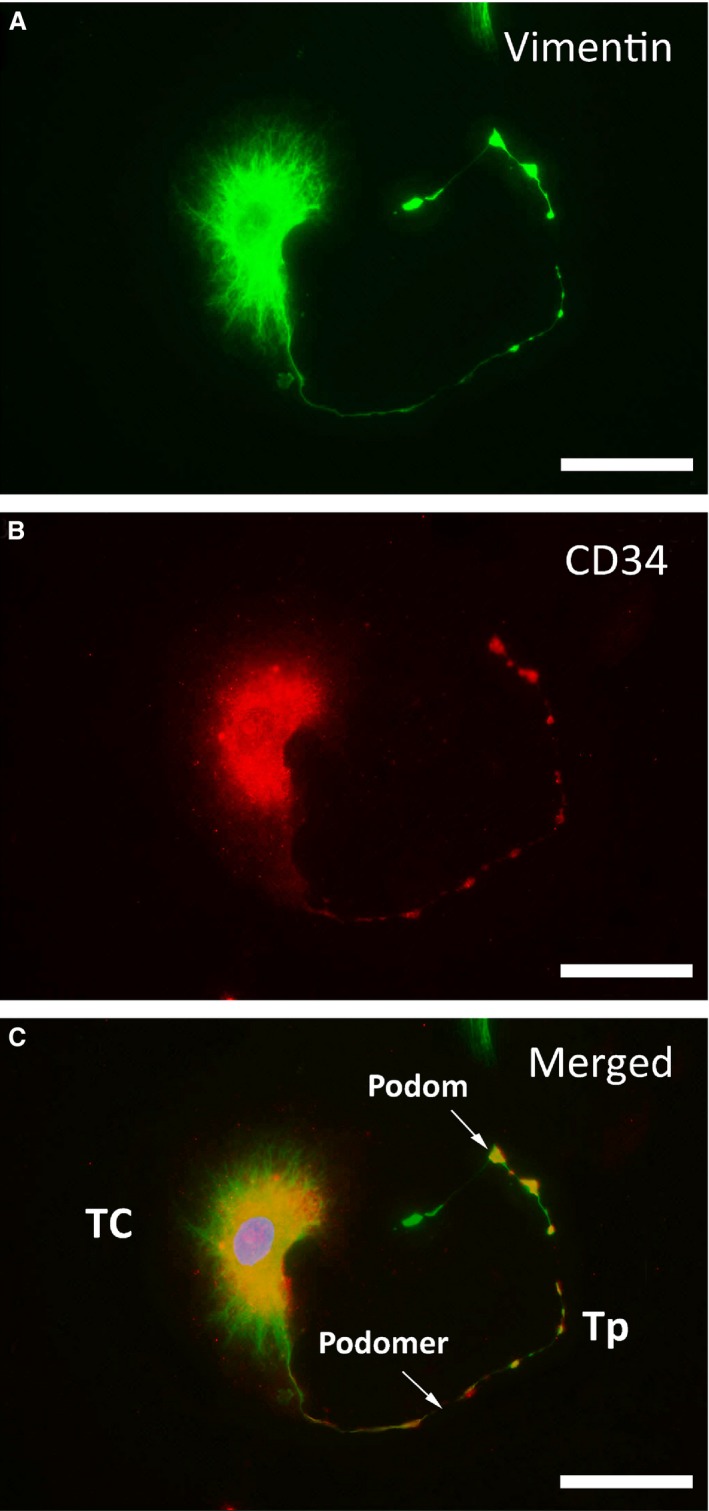
Representative double‐labelled immunofluorescence images. Nuclei were counterstained with DAPI (blue). Images of negative staining for c‐kit are not shown; scale bar = 50 μm. (**A**) Positive FITC labelling for vimentin (green). (**B**) Positive CY3 labelling for CD34 (red). (**C**) In the merged image, vimentin and CD34 are co‐localized in TCs, both in the cellular body and in Tps. Podomers and podoms are arrayed along Tps.

Telocytes were stained for vimentin and CD34, and a significant number of vimentin‐positive cells (green) also exhibited CD34‐positive fluorescence (red). Furthermore, the alternating thick and thin segments of Tps could be clearly identified (Fig. 2). However, no fluorescence for c‐Kit (images not shown) was observed.

### Morphological study of pMACs

**Figure 3:**
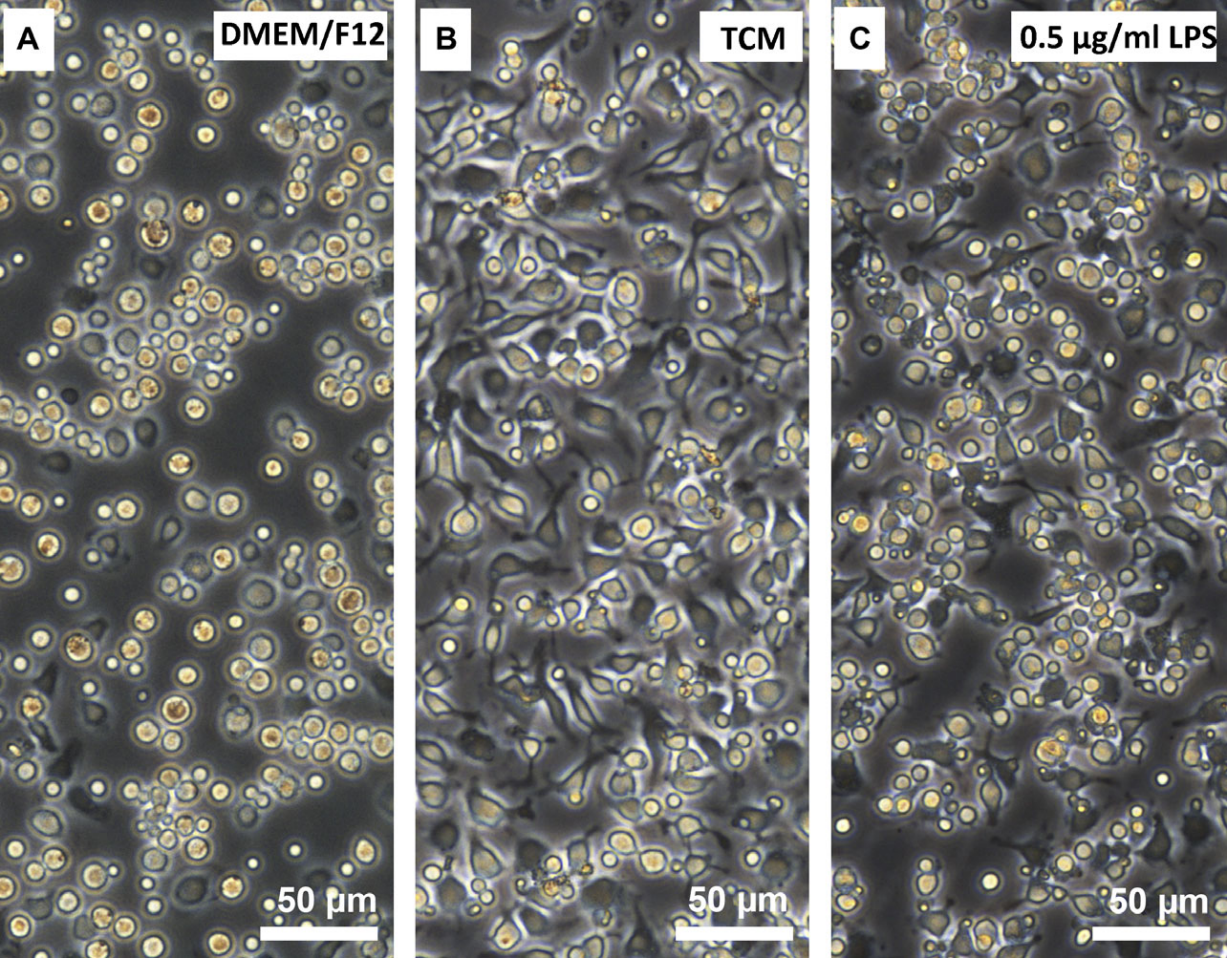
Morphological alterations in mouse pMACs following exposure to TCM, DMEM/F12, or LPS for 48 hrs. (**A**) DMEM/F12‐treated pMACs showed no signs of activation: they had normal morphology with a regular round shape, abundant clear cytoplasm and ample intercellular spaces. (**B**) TCM‐treated pMACs had a relatively moderate activation/immune response: they exhibited obvious morphological changes with a polyhedron shape, large and sufficient pseudopodia, abundant granules in the cytoplasm, narrow intercellular spaces and densely populated features. (**C**) LPS (0.5 μg/ml)‐treated pMACs showed excessive activation: they presented with irregular, doublet or multiple shapes and ultimately underwent cell death, which was characterized by cell membrane blebbing, cell body atrophy and nuclear condensation or fragmentation.

After 48 hrs of co‐culture with TCM, DMEM/F12, or LPS (0.5 μg/ml), the morphological changes in pMACs were obviously different (Fig. 3). DMEM/F12‐treated pMACs showed no obvious morphological abnormalities and no sign of activation or an immunoresponse (Fig. 3A). Conversely, TCM‐treated pMACs contained abundant pseudopodia and secretory granules within the cytoplasm, with no obvious cell death after 48 hrs, suggesting a relatively moderate activation/immunoresponse (Fig. 3B). However, more dramatic morphological changes were observed after treatment with LPS, and these changes were accompanied by obvious cell death at 24 hrs, indicating excessive activation or over‐stimulation of pMACs (Fig. 3C).

### Cell viability of pMACs

**Figure 4:**
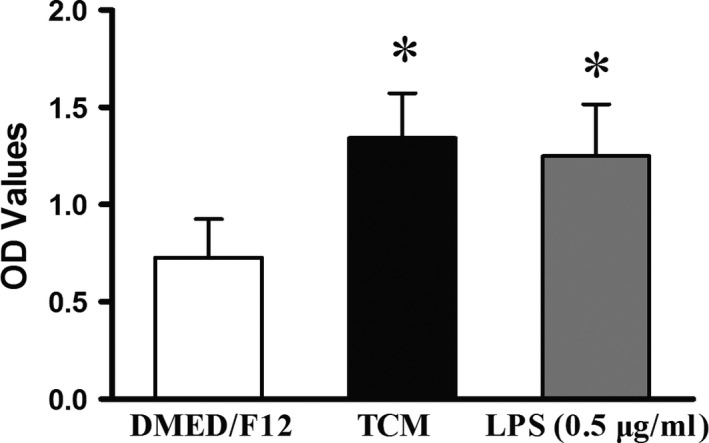
Cell viability of pMACs after 48 hrs of co‐culture. As demonstrated by increased OD values, TCM and LPS significantly activated pMACs compared to DMEM/F12. Nevertheless, no significant difference was observed between TCM and LPS (*P* > 0.05), although a slightly higher value was obtained for TCM. *P < 0.05 *versus* DMEM/F12; error bars = SD. The data are representative of at least 9 values from three independent experiments.

One‐way anova and Dunnett's test revealed a significant difference between the TCM and DMEM/F12 treatments as well as between the LPS and DMEM/F12 treatments (both *P* < 0.05) in terms of their effect on cell viability. However, the slightly higher viability observed for TCM‐treated pMACs compared to LPS‐treated pMACs was not significant (*P* > 0.05), indicating that TCM and LPS efficiently activated pMACs to the same extent (Fig. 4).

### Quantitative analysis of cytokines/enzymes

**Figure 5:**
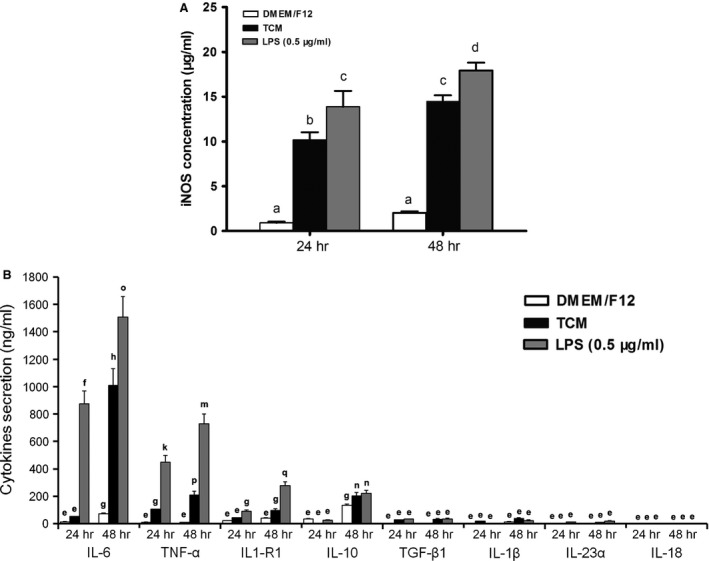
Quantitative analysis of nine cytokines/enzymes secreted by pMACs after exposure to TCM, DMEM/F12 or LPS (0.5 μg/ml) for 24 or 48 hrs. The mean and SD were calculated from nine values from three independent experiments. The bars that do not share a letter represent data that are significantly different (*P *<* *0.05). (**A** and **B**) The concentrations of iNOS and IL‐6 were significantly elevated, and TNF‐α, IL1‐R1 and IL‐10 levels were slightly, but significantly, increased. At 24 or 48 hrs, one‐way anova revealed significant differences in iNOS, IL‐6, TNF‐α, IL1‐R1 and IL‐10 levels after treatment with TCM, DMEM/F12 or LPS (all *P* < 0.05). Dunnett's test showed significant differences between DMEM/F12 and the test values (all *P* < 0.05). Student's *t*‐test revealed significant differences in the TCM and LPS groups at the 24 and 48 hrs time points (all *P* < 0.05). (**B**) No significant changes in TGF‐β1, IL‐1β, IL‐23α and IL‐18 were observed. The bars that share a letter were either not significantly different (*P* > 0.05), or their values were too low for biological significance.

As indicated by one‐way anova or Student's *t*‐test followed by Dunnett's test, iNOS and IL‐6 were significantly increased in TCM‐treated pMACs compared to DMEM/F12‐treated pMACs at 24 and/or 48 hrs, but the levels of these proteins were always higher in LPS‐treated cells (all *P* < 0.05; Fig. 5A and B). Meanwhile, slightly but significantly increased levels of TNF‐α, IL1‐R1, and IL‐10 were observed in TCM‐treated pMACs, but again, these levels were lower than those in the LPS‐treated cells at the two time points (all *P* < 0.05). However, no obvious fluctuations in TGF‐β1, IL‐1β, IL‐23α and IL‐18 levels were observed throughout the experiment (Fig. 5B).

## Discussion

Recent studies have identified new cellular elements in female reproductive organs that are now named TCs 1, 2, 3, 4, 5, 6, 7, 8, 9, 10, 11, 12, 13, 14, 15, 16, 17, 18, 19, 20, 21, 22, 23, 24, 25. Multiple functions have been proposed for uterine TCs, including in modulating the contraction of the myometrium 5, 13, 16, 17, 22, 24 and regulating the blood volume in the placenta 2, 10, 19, 25 and the self‐renewal of the endometrium or myometrium 4. However, the connectivity between TCs and multiple immunocytes in normal tissue 7, 8, 9, 10, 44, 45, 46, 47, 48, 49, 50, 51, 52, 53, 54, 55, 56, 72 and in inflammation‐affected oviduct tissue from an SD rat model 11, 12 evoked the hypothesis that TCs might act as antigen processing cells or local data suppliers, thereby promoting signal transduction and triggering immunocyte migration and the immune response (repression or activation). Nevertheless, no reliable *in vitro* evidence is currently available to support an immunoregulatory/immunosurveillance role for TCs.

To confirm the aforementioned *in vivo* findings, primary mouse uterine TCs were cultured, and typical TCs were confirmed both structurally and phenotypically. However, the immunophenotype of mouse uterine TCs varies among reports, possibly because of the existence of different subpopulations of uterine TCs 6. Peritoneal macrophages, one of the most important peritoneal immunocytes related to endometriosis, pelvic pain and infertility 73, were also harvested for a co‐culture study. Multiple morphological changes occurred after culture with either TCM or LPS, generating a similar appearance to macrophages responding to infectious pathogens 74, 75. However, LPS‐treated pMACs underwent more dramatic morphological changes than TCM‐treated cells. This was further demonstrated by the CCK‐8 viability analysis and the overproduction of a panel of macrophage‐derived cytokines/enzyme in the TCM and LPS groups. Peritoneal macrophages are terminally differentiated cells that do not have the potential to proliferate but do have the capacity to activate the immune response. Therefore, we report for the first time that TCM demonstrated the ability to activate pMACs and potentially trigger a subsequent immune response that was similar to, but slightly weaker than, that elicited by the classical stimulus, LPS. We believe that the panel of elevated cytokines/enzymes plays an important role in reproductive physiology.

Inducible nitric oxide synthase, an enzyme that catalyses the production of nitric oxide from L‐arginine upon stimulation by pro‐inflammatory cytokines (*e.g*., IL‐1, tumour necrosis factor α and interferon γ), has been suggested to participate in host immunity and anti‐microbial and anti‐tumour activities as part of the oxidative burst of macrophages 76. Moreover, the pathological generation of nitric oxide through increased iNOS activity may decrease the tubal ciliary beat frequency and oviductal smooth muscle activity, further affecting the intra‐tubal transport of ova or fertilized eggs, which might consequently result in a tubal ectopic pregnancy or tubal factor infertility and most likely causes uterine contractility disorders 77. In the uterus, the local production of nitric oxide plays a role in uterine cyclicity 78, participates in growth associated with the decidual response 79, and reflects embryonic health 80. Generally, elevated nitric oxide values are critical for successful embryo implantation during early pregnancy by promoting vasodilation and smooth muscle relaxation 81. In contrast, nitric oxide production is suppressed in the later stage of gestation to prepare the uterus for parturition 82. Therefore, a balanced iNOS/nitric oxide system is critical for successful early implantation, pregnancy and labor. However, TCM‐induced elevations in iNOS levels might cause several reproductive abnormalities.

Interleukin‐6, a multifunctional cytokine mainly produced by antigen‐processing cells such as macrophages and B cells, functions as a regulator of the immune response and local acute inflammation. IL‐6 has broad functional range, affecting the renewal of endometrial blood vessels and menstrual shedding as well as preparing the endometrium for early embryo implantation events, maturation and expansion of foetal precursors 83, 84. Furthermore, IL‐6 is a physiological mediator of the expression of other genes that have documented or presumed roles in pregnancy, such as stimulating the release of human chorionic gonadotropin from syncytiotrophoblast cells 85, 86. Increased concentrations of IL‐6 are linked to proliferative disorders of the endometrium, such as endometriosis 87. Nevertheless, excessive IL‐6 levels induced by TCM might cause an improper endometrial state and implantation failure.

Tumour necrosis factor α, originally known for its tumour cytotoxicity, is a potent mediator of inflammation. Normal levels of TNF‐α in the uterus contribute to cell adhesion, hormone production and cyclic remodelling of the endometrium, which is mediated by the modulation of endometrial cells and promotes neovascularization 88, 89. Tumour necrosis factor α also prevents inappropriate trophoblast cell invasion into the uterus and promotes the growth of embryonic mesenchymal cells 90, 91. However, the over‐expression of TNF‐α not only stimulates the production of nitric oxide but also causes pathophysiological effects, such as implantation failure and immunologically mediated abortion.

Interleukin‐1R1, one of two known receptors for IL‐1, modulates endometrial receptivity, creates a receptive endometrial epithelium and represents a key molecule for successful implantation 92, 93. Strong expression of IL‐1R1 is related to the risk of developing endometriosis 94. Therefore, the up‐regulation of IL‐1R1 by TCM might create an abnormal peri‐implantation environment and lead to blastocyst implantation failure.

Interleukin‐10 is expressed in the placenta in a gestational age‐dependent manner, and its down‐regulation at term is an important mechanism associated with parturition 95. Interleukin‐10 plays positive roles in protecting pregnancy by decreasing trophoblast cell apoptosis, inflammation and endothelial cell dysfunction; IL‐10 deficiency potentially causes adverse pregnancy outcome and foetal death 96, 97. Therefore, IL‐10 has been considered an essential molecule in the maintenance of a successful pregnancy. However, excessive IL‐10 production in response to TCM might lead to an adverse pregnancy outcome.

Nevertheless, more questions remain, including what mechanism is responsible for the elevated levels of iNOS, IL‐6, TNF‐α, IL‐1R1 and IL‐10. Generally, TCs release at least three types of EVs: exosomes, ectosomes and multivesicular bodies, which contain many secretomes 4, 5, 35, 36, 37, 38, 39, 40, 41, 42, 43, 44, 45, 46, 63, 64, 65, 66, 71. Such essential paracrine mediators protect, enrich and transfer complex multimolecular biological messages from EVs within TCs and Tps, thus helping to achieve the immunoregulatory/immunosurveillance roles of TCM. Notably, supernatants from primary human lung and esophagus TC cultures contained increased concentrations of both VEGF and EGF 47, 57. Additionally, transplanting renal TCs significantly increased the local mRNA levels of several growth factors, including HGF, EGF, PDGF and IGF‐1 60. More recently, the TC secretome was found to contain a panel of chemokines, cytokines and growth factors 71. Thus, these data provide evidence of paracrine secretion by TCs and suggest their possible role in cell proliferation, differentiation and tissue repair.

Nuclear Factor kappa B (NF‐κB) is the most common downstream effector in pMAC activation. Its activation and phosphorylation leads to an enhanced immuno‐inflammatory response and the synthesis of numerous inflammatory cytokines. Transplanting renal TCs activated the NF‐κB signalling pathway in a renal ischaemia‐reperfusion injury model and up‐regulated the mRNA levels of pro‐inflammatory cytokines, such as IL‐1 and TNF‐α 60. However, the exact pathway and the complex networks responsible for the overproduction of cytokines/enzymes, as well as the crosstalk or reciprocal influence between pMACs and TCM, remain to be fully elucidated.

## Conclusion

Our results provide the first preliminary *in vitro* evidence that TCs are not innocent bystanders but are instead potential active players in the induction and maintenance of the inflammatory process. These data support our previously proposed hypothesis that TCs participate in local immunoregulation/immunosurveillance. Meanwhile, it remains unknown whether this immuno‐inflammatory response is localized to only uterine TCs or is systemic. Additionally, it remains to be determined whether the observed modulatory effects of low‐level laser stimulation on TC growth 20 represents a novel therapy for TC‐associated abnormalities related to local immunity. Nevertheless, in future studies, animal models must be designed to better clarify the functional consequences and clinical applications of TCs in immune‐mediated fertility problems and other related diseases, thereby strengthening the proposed immunoregulatory/immunosurveillance roles of TCs.

## Conflicts of interest

The authors confirm that there are no conflicts of interest.

## References

[1] Popescu LM , Ciontea SM , Cretoiu D . Interstitial Cajal‐like cells in human uterus and fallopian tube. Ann N Y Acad Sci. 2007; 1101: 139–65. 17360808 10.1196/annals.1389.022

[2] Suciu L , Popescu LM , Gherghiceanu M , *et al* Telocytes in human term placenta: morphology and phenotype. Cells Tissues Organs. 2010; 192: 325–39. 20664249 10.1159/000319467

[3] Cretoiu SM , Cretoiu D , Suciu L , *et al* Interstitial Cajal‐like cells of human Fallopian tube express estrogen and progesterone receptors. J Mol Histol. 2009; 40: 387–94. 20063045 10.1007/s10735-009-9252-z

[4] Creţoiu SM , Creţoiu D , Popescu LM . Human myometrium–the ultrastructural 3D network of telocytes. J Cell Mol Med. 2012; 16: 2844–9. 23009098 10.1111/j.1582-4934.2012.01651.x PMC4118253

[5] Cretoiu SM , Cretoiu D , Marin A , *et al* Telocytes: ultrastructural, immunohistochemical and electrophysiological characteristics in human myometrium. Reproduction. 2013; 145: 357–70. 23404846 10.1530/REP-12-0369 PMC3636525

[6] Ciontea SM , Radu E , Regalia T , *et al* C‐kit immunopositive interstitial cells (Cajal‐type) in human myometrium. J Cell Mol Med. 2005; 9: 407. 15963260 10.1111/j.1582-4934.2005.tb00366.x PMC6740058

[7] Ullah S , Yang P , Zhang L , *et al* Identification and characterization of telocytes in the uterus of the oviduct in the Chinese soft‐shelled turtle, *Pelodiscus sinensis*: TEM evidence. J Cell Mol Med. 2014; 18: 2385–92. 25230849 10.1111/jcmm.12392 PMC4302644

[8] Cretoiu SM , Cretoiu D , Simionescu AA , *et al* Telocytes in human fallopian tube and uterus express estrogen and progesterone receptors. Sex Steroids. 2012; 217: 91–114.

[9] Popescu LM , Ciontea SM , Cretoiu D , *et al* Novel type of interstitial cell (Cajal‐like) in human fallopian tube. J Cell Mol Med. 2005; 9: 479–523. 15963270 10.1111/j.1582-4934.2005.tb00376.x PMC6740321

[10] Bosco C , Díaz E , Gutiérrez R , *et al* A putative role for telocytes in placental barrier impairment during preeclampsia. Med Hypotheses. 2015; 84: 72–7. 25499002 10.1016/j.mehy.2014.11.019

[11] Yang XJ , Yang J , Liu Z , *et al* Telocytes damage in endometriosis‐affected rat oviduct and potential impact on fertility. J Cell Mol Med. 2015; 19: 452–62. 25388530 10.1111/jcmm.12427 PMC4407595

[12] Yang J , Chi C , Liu Z , *et al* Ultrastructure damage of oviduct telocytes in rat model of acute salpingitis. J Cell Mol Med. 2015; 19: 1720–8. 25753567 10.1111/jcmm.12548 PMC4511368

[13] Cretoiu S , Simionescu A , Caravia L , *et al* Complex effects of imatinib on spontaneous and oxytocin‐induced contractions in human non‐pregnant myometrium. Acta Physiol Hung. 2011; 98: 329–38. 21893472 10.1556/APhysiol.98.2011.3.10

[14] Popescu LM , Vidulescu C , Curici A , *et al* Imatinib inhibits spontaneous rhythmic contractions of human uterus and intestine. Eur J Pharmacol. 2006; 546: 177–81. 16919263 10.1016/j.ejphar.2006.06.068

[15] Gravina FS , van Helden DF , Kerr KP , *et al* Phasic contractions of the mouse vagina and cervix at different phases of the estrus cycle and during late pregnancy. PLoS ONE. 2014; 9: e111307. 25337931 10.1371/journal.pone.0111307 PMC4206458

[16] Duquette RA , Shmygol A , Vaillant C , *et al* Vimentin‐positive, c‐kit‐negative interstitial cells in human and rat uterus: a role in pacemaking? Biol Reprod. 2005; 72: 276–83. 15385413 10.1095/biolreprod.104.033506

[17] Allix S , Reyes‐Gomez E , Aubin‐Houzelstein G , *et al* Uterine contractions depend on KIT‐positive interstitial cells in the mouse: genetic and pharmacological evidence. Biol Reprod. 2008; 79: 510–7. 18480468 10.1095/biolreprod.107.066373

[18] Cretoiu D , Ciontea SM , Popescu LM , *et al* Interstitial Cajal‐like cells (ICLC) as steroid hormone sensors in human myometrium: immunocytochemical approach. J Cell Mol Med. 2006; 10: 789–95. 16989738 10.1111/j.1582-4934.2006.tb00438.x PMC3933160

[19] Suciu L , Popescu LM , Gherghiceanu M . Human placenta: *de visu* demonstration of interstitial Cajal‐like cells. J Cell Mol Med. 2007; 11: 590–7. 17635651 10.1111/j.1582-4934.2007.00058.x PMC3922366

[20] Campeanu RA , Radu BM , Cretoiu SM , *et al* Near‐infrared low‐level laser stimulation of telocytes from human myometrium. Laser Med Sci. 2014; 29: 1867–74. 10.1007/s10103-014-1589-1 PMC4215113 24870411

[21] Popescu LM , Andrei F , Hinescu ME . Snapshots of mammary gland interstitial cells: methylene‐blue vital staining and c‐kit immunopositivity. J Cell Mol Med. 2005; 9: 476–7. 15963269 10.1111/j.1582-4934.2005.tb00375.x PMC6740295

[22] Rosenbaum ST , Svalø J , Nielsen K , *et al* Immunolocalization and expression of small‐conductance calcium‐activated potassium channels in human myometrium. J Cell Mol Med. 2012; 16: 3001–8. 22947283 10.1111/j.1582-4934.2012.01627.x PMC4393728

[23] Hatta K , Huang ML , Weisel RD , *et al* Culture of rat endometrial telocytes. J Cell Mol Med. 2012; 16: 1392–6. 22551155 10.1111/j.1582-4934.2012.01583.x PMC3823209

[24] Cretoiu SM , Radu BM , Banciu A , *et al* Isolated human uterine telocytes: immunocytochemistry and electrophysiology of T‐type calcium channels. Histochem Cell Biol. 2015; 143: 83–94. 25212658 10.1007/s00418-014-1268-0 PMC4286651

[25] Bosco C , Díaz E , Gutiérrez R , *et al* Placental hypoxia developed during preeclampsia induces telocytes apoptosis in chorionic villi affecting the maternal‐fetus metabolic exchange. Curr Stem Cell Res Ther. 2015; Doi:10.2174/1574888X10666150202144855. 10.2174/1574888x10666150202144855 25643124

[26] Popescu LM , Faussone‐Pellegrini MS . TELOCYTES–a case of serendipity: the winding way from Interstitial Cells of Cajal (ICC), *via* Interstitial Cajal‐Like Cells (ICLC) to TELOCYTES. J Cell Mol Med. 2010; 14: 729–40. 20367664 10.1111/j.1582-4934.2010.01059.x PMC3823108

[27] Popescu LM , Manole CG , Gherghiceanu M , *et al* Telocytes in human epicardium. J Cell Mol Med. 2010; 14: 2085–93. 20629996 10.1111/j.1582-4934.2010.01129.x PMC3823000

[28] Manole CG , Cismaşiu V , Gherghiceanu M , *et al* Experimental acute myocardial infarction: telocytes involvement in neo‐angiogenesis. J Cell Mol Med. 2011; 15: 2284–96. 21895968 10.1111/j.1582-4934.2011.01449.x PMC3822940

[29] Hinescu ME , Popescu LM . Interstitial Cajal‐like cells (ICLC) in human atrial myocardium. J Cell Mol Med. 2005; 9: 972–5. 16364205 10.1111/j.1582-4934.2005.tb00394.x PMC6740218

[30] Hinescu ME , Gherghiceanu M , Suciu L , *et al* Telocytes in pleura: two‐and three‐dimensional imaging by transmission electron microscopy. Cell Tissue Res. 2011; 343: 389–97. 21174125 10.1007/s00441-010-1095-0 PMC3032227

[31] Zheng Y , Li H , Manole C , *et al* Telocytes in trachea and lungs. J Cell Mol Med. 2011; 15: 2262–8. 21810171 10.1111/j.1582-4934.2011.01404.x PMC4394233

[32] Kostin S . Myocardial telocytes: a specific new cellular entity. J Cell Mol Med. 2010; 14: 1917–21. 20604817 10.1111/j.1582-4934.2010.01111.x PMC3823273

[33] Edelstein L , Smythies J . The role of telocytes in morphogenetic bioelectrical signaling: once more unto the breach. Front Mol Neurosci. 2014; 7: 41. 24860423 10.3389/fnmol.2014.00041 PMC4026729

[34] Ceafalan L , Gherghiceanu M , Popescu LM , *et al* Telocytes in human skin–are they involved in skin regeneration? J Cell Mol Med. 2012; 16: 1405–20. 22500885 10.1111/j.1582-4934.2012.01580.x PMC3823211

[35] Smythies J , Edelstein L . Telocytes, exosomes, gap junctions and the cytoskeleton: the makings of a primitive nervous system? Front Cell Neurosci. 2014; 7: 278. 24427115 10.3389/fncel.2013.00278 PMC3879459

[36] Nicolescu MI , Popescu LM . Telocytes in the interstitium of human exocrine pancreas: ultrastructural evidence. Pancreas. 2012; 41: 949–56. 22318257 10.1097/MPA.0b013e31823fbded

[37] Fertig ET , Gherghiceanu M , Popescu LM . Extracellular vesicles release by cardiac telocytes: electron microscopy and electron tomography. J Cell Mol Med. 2014; 18: 1938–43. 25257228 10.1111/jcmm.12436 PMC4244009

[38] Mandache E , Popescu LM , Gherghiceanu M . Myocardial interstitial Cajal‐like cells (ICLC) and their nanostructural relationships with intercalated discs: shed vesicles as intermediates. J Cell Mol Med. 2007; 11: 1175–84. 17979892 10.1111/j.1582-4934.2007.00117.x PMC4401282

[39] Popescu LM , Gherghiceanu M , Suciu LC , *et al* Telocytes and putative stem cells in the lungs: electron microscopy, electron tomography and laser scanning microscopy. Cell Tissue Res. 2011; 345: 391–403. 21858462 10.1007/s00441-011-1229-z PMC3168741

[40] Rusu MC , Mirancea N , Mănoiu VS , *et al* Skin telocytes. Ann Anat. 2012; 194: 359–67. 22226149 10.1016/j.aanat.2011.11.007

[41] Cismasiu VB , Popescu LM . Telocytes transfer extracellular vesicles loaded with microRNAs to stem cells. J Cell Mol Med. 2015; 19: 351–8. 25600068 10.1111/jcmm.12529 PMC4407601

[42] Bei Y , Wang F , Yang C , *et al* Telocytes in regenerative medicine. J Cell Mol Med. 2015; 19: 1441–54. 26059693 10.1111/jcmm.12594 PMC4511344

[43] Roatesi I , Radu BM , Cretoiu D , *et al* Uterine telocytes: a review of current knowledge. Biol Reprod. 2015; 93: 1–13; Doi:10.1095/biolreprod.114.125906. 10.1095/biolreprod.114.125906 25695721

[44] Cretoiu SM , Popescu LM . Telocytes revisited. Biomol Concepts. 2014; 5: 353–69. 25367617 10.1515/bmc-2014-0029

[45] Nicolescu MI , Bucur A , Dinca O , *et al* Telocytes in parotid glands. Anat Rec. 2012; 295: 378–85. 10.1002/ar.21540 22174191

[46] Popescu LM , Gherghiceanu M , Cretoiu D , *et al* The connective connection: interstitial cells of Cajal (ICC) and ICC‐like cells establish synapses with immunoreactive cells: electron microscope study in sity. J Cell Mol Med. 2005; 9: 714–30. 16202219 10.1111/j.1582-4934.2005.tb00502.x PMC6741637

[47] Chen X , Zheng Y , Manole CG , *et al* Telocytes in human oesophagus. J Cell Mol Med. 2013; 17: 1506–12. 24188731 10.1111/jcmm.12149 PMC4117563

[48] Luesma MJ , Gherghiceanu M , Popescu LM . Telocytes and stem cells in limbus and uvea of mouse eye. J Cell Mol Med. 2013; 17: 1016–24. 23991685 10.1111/jcmm.12111 PMC3780542

[49] Popescu BO , Gherghiceanu M , Kostin S , *et al* Telocytes in meninges and choroid plexus. Neurosci Lett. 2012; 516: 265–9. 22516459 10.1016/j.neulet.2012.04.006

[50] Manetti M , Guiducci S , Ruffo M , *et al* Evidence for progressive reduction and loss of telocytes in the dermal cellular network of systemic sclerosis. J Cell Mol Med. 2013; 17: 482–96. 23444845 10.1111/jcmm.12028 PMC3822649

[51] Cretoiu D , Cretoiu SM , Simionescu AA , *et al* Telocytes, a distinct type of cell among the stromal cells present in the lamina propria of jejunum. Histol Histopathol. 2012; 27: 1067–78. 22763879 10.14670/HH-27.1067

[52] Alunno A , Ibba‐Manneschi L , Bistoni O , *et al* Telocytes in minor salivary glands of primary Sjögren's syndrome: association with the extent of inflammation and ectopic lymphoid neogenesis. J Cell Mol Med. 2015; 19: 1689–96. 25753463 10.1111/jcmm.12545 PMC4511365

[53] Milia AF , Ruffo M , Manetti M , *et al* Telocytes in Crohn's disease. J Cell Mol Med. 2013; 17: 1525–36. 24251911 10.1111/jcmm.12177 PMC3914651

[54] Manetti M , Rosa I , Messerini L , *et al* Telocytes are reduced during fibrotic remodelling of the colonic wall in ulcerative colitis. J Cell Mol Med. 2015; 19: 62–73. 25283476 10.1111/jcmm.12457 PMC4288350

[55] Cantarero Carmona I , Luesma Bartolomé MJ , Junquera Escribano C . Identification of telocytes in the lamina propria of rat duodenum: transmission electron microscopy. J Cell Mol Med. 2011; 15: 26–30. 21054782 10.1111/j.1582-4934.2010.01207.x PMC3822490

[56] Manole CG , Gherghiceanu M , Simionescu O . Telocyte dynamics in psoriasis. J Cell Mol Med. 2015; 19: 1504–19. 25991475 10.1111/jcmm.12601 PMC4511349

[57] Zheng Y , Chen X , Qian M , *et al* Human lung telocytes could promote the proliferation and angiogenesis of human pulmonary microvascular endothelial cells *in vitro* . Mol Cell Ther. 2014; 2: 3. 26056572 10.1186/2052-8426-2-3 PMC4452074

[58] Mou Y , Wang Y , Li J , *et al* Immunohistochemical characterization and functional identification of mammary gland telocytes in the self‐assembly of reconstituted breast cancer tissue *in vitro* . J Cell Mol Med. 2013; 17: 65–75. 23206234 10.1111/j.1582-4934.2012.01646.x PMC3823137

[59] Gherghiceanu M , Popescu LM . Interstitial Cajal‐like cells (ICLC) in human resting mammary gland stroma. Transmission electron microscope (TEM) identification. J Cell Mol Med. 2005; 9: 893–910. 16364198 10.1111/j.1582-4934.2005.tb00387.x PMC6740089

[60] Li L , Lin M , Li L , *et al* Renal telocytes contribute to the repair of ischemically injured renal tubules. J Cell Mol Med. 2014; 18: 1144–56. 24758589 10.1111/jcmm.12274 PMC4508154

[61] Popescu LM . The tandem: telocytes–stem cells. Int J Biol Biomed Eng. 2011; 5: 83–92.

[62] Diaz‐Flores L , Gutierrez R , Garcia MP , *et al* CD34^+^ stromal cells/fibroblasts/fibrocytes/telocytes as a tissue reserve and a principal source of mesenchymal cells. Location, morphology, function and role in pathology. Histol Histopathol. 2014; 29: 831–70. 24488810 10.14670/HH-29.831

[63] Zheng Y , Cretoiu D , Yan G , *et al* Protein profiling of human lung telocytes and microvascular endothelial cells using iTRAQ quantitative proteomics. J Cell Mol Med. 2014; 18: 1035–59. 25059386 10.1111/jcmm.12350 PMC4508144

[64] Popescu LM , Manole E , Şerboiu CS , *et al* Identification of telocytes in skeletal muscle interstitium: implication for muscle regeneration. J Cell Mol Med. 2011; 15: 1379–92. 21609392 10.1111/j.1582-4934.2011.01330.x PMC4373336

[65] Popescu LM , Nicolescu MI . Telocytes and stem cells In: Goldenberg RCdS, deCampos Carvalho AC, editors. Resident stem cells and regenerative therapy. Oxford: Academic Press/Elsevier; 2013 pp. 205–31.

[66] Faussone‐Pellegrini MS , Popescu LM . Telocytes. Biomol Concepts. 2011; 2: 481–9. 25962049 10.1515/BMC.2011.039

[67] Gevaert T , Vos R , Aa F , *et al* Identification of telocytes in the upper lamina propria of the human urinary tract. J Cell Mol Med. 2012; 16: 2085–93. 22151349 10.1111/j.1582-4934.2011.01504.x PMC3822978

[68] Gherghiceanu M , Popescu LM . Cardiac telocytes—their junctions and functional implications. Cell Tissue Res. 2012; 348: 265–79. 22350946 10.1007/s00441-012-1333-8 PMC3349856

[69] Cismasiu VB , Radu E , Popescu LM . miR‐193 expression differentiates telocytes from other stromal cells. J Cell Mol Med. 2011; 15: 1071–4. 21447044 10.1111/j.1582-4934.2011.01325.x PMC3822620

[70] Zhao B , Chen S , Liu J , *et al* Cardiac telocytes were decreased during myocardial infarction and their therapeutic effects for ischaemic heart in rat. J Cell Mol Med. 2013; 17: 123–33. 23205601 10.1111/j.1582-4934.2012.01655.x PMC3823142

[71] Albulescu R , Tanase C , Codrici E , *et al* The secretome of myocardial telocytes modulates the activity of cardiac stem cells. J Cell Mol Med. 2015; 19: 1783–94. Doi:10.1111/jcmm.12624. 26176909 10.1111/jcmm.12624 PMC4549029

[72] Manetti M , Rosa I , Messerini L , *et al* A loss of telocytes accompanies fibrosis of multiple organs in systemic sclerosis. J Cell Mol Med. 2014; 18: 253–62. 24467430 10.1111/jcmm.12228 PMC3930412

[73] Tran LV , Tokushige N , Berbic M , *et al* Macrophages and nerve fibres in peritoneal endometriosis. Hum Reprod. 2009; 1: 1–7. 10.1093/humrep/den483 19136478

[74] Savill JS , Wyllie AH , Henson JE , *et al* Macrophage phagocytosis of aging neutrophils in inflammation. Programmed cell death in the neutrophil leads to its recognition by macrophages. J Clin Invest. 1989; 83: 865. 2921324 10.1172/JCI113970 PMC303760

[75] Porcheray F , Viaud S , Rimaniol AC , *et al* Macrophage activation switching: an asset for the resolution of inflammation. Clin Exp Immunol. 2005; 142: 481–9. 16297160 10.1111/j.1365-2249.2005.02934.x PMC1809537

[76] Moncada S , Palmer RM , Higgs EA . Nitric oxide: physiology, pathophysiology, and pharmacology. Pharmacol Rev. 1991; 43: 109–42. 1852778

[77] Martinez SP , Viggiano M , Franchi AM , *et al* Effect of nitric oxide synthase inhibitors on ovum transport and oviductal smooth muscle activity in the rat oviduct. J Reprod Fertil. 2000; 118: 111–7. 10793632 10.1530/jrf.0.1180111

[78] Huang J , Roby KF , Pace JL , *et al* Cellular localization and hormonal regulation of inducible nitric oxide synthase in cycling mouse uterus. J Leukocyte Biol. 1995; 57: 27–35. 7530281 10.1002/jlb.57.1.27

[79] Spencer F , Chi L , Zhu MX . Antiproliferative effects of inducible nitric oxide synthase inhibition on decidualization in pseudopregnant rats. Exp Biol Med. 1998; 218: 45–50. 10.3181/00379727-218-44266 9572151

[80] Khorram O . Nitric oxide and its role in blastocyst implantation. Rev Endocr Metab Dis. 2002; 3: 145–9. 10.1023/a:1015459029397 12007291

[81] Purcell TL , Given R , Chwalisz K , *et al* Nitric oxide synthase distribution during implantation in the mouse. Mol Hum Reprod. 1999; 5: 467–75. 10338370 10.1093/molehr/5.5.467

[82] Thomson AJ , Telfer JF , Kohnen G , *et al* Nitric oxide synthase activity and localization do not change in uterus and placenta during human parturition. Hum Reprod. 1997; 12: 2546–52. 9436704 10.1093/humrep/12.11.2546

[83] Smith SK , Charnock‐Jones DS , Sharkey AM . The role of leukaemia inhibitory factor and interleukin‐6 in human reproduction. Hum Reprod. 1998; 13: 237–43. 9755426 10.1093/humrep/13.suppl_3.237

[84] Laird SM , Tuckerman EM , Cork BA , *et al* Expression of nuclear factor κB in human endometrium; role in the control of interleukin 6 and leukaemia inhibitory factor production. Mol Hum Reprod. 2000; 6: 34–40. 10611258 10.1093/molehr/6.1.34

[85] Modrić T , Kowalski AA , Green ML , *et al* Pregnancy‐dependent expression of leukaemia inhibitory factor (LIF), LIF receptor‐β and interleukin‐6 (IL‐6) messenger ribonucleic acids in the porcine female reproductive tract. Placenta. 2000; 21: 345–53. 10833369 10.1053/plac.1999.0493

[86] Stephanou A , Handwerger S . Interleukin‐6 stimulates placental lactogen expression by human trophoblast cells. Endocrinology. 1994; 135: 719–23. 8033820 10.1210/endo.135.2.8033820

[87] Zarmakoupis PN , Rier SE , Maroulis GB , *et al* Uterus and endometrium: inhibition of human endometrial stromal cell proliferation by interleukin 6. Hum Reprod. 1995; 10: 2395–9. 8530673 10.1093/oxfordjournals.humrep.a136306

[88] Von Wolff M , Classen‐Linke I , Heid D , *et al* Tumour necrosis factor‐α (TNF‐α) in human endometrium and uterine secretion: an evaluation by immunohistochemistry, ELISA and semiquantitative RT–PCR. Mol Hum Reprod. 1999; 5: 146–52. 10065870 10.1093/molehr/5.2.146

[89] Hunt JS , Chen HL , Miller L . Tumor necrosis factors: pivotal components of pregnancy? Biol Reprod. 1996; 54: 554–62. 8835376 10.1095/biolreprod54.3.554

[90] Chen HL , Yang YP , Hu XL , *et al* Tumor necrosis factor alpha mRNA and protein are present in human placental and uterine cells at early and late stages of gestation. Am J Pathol. 1991; 139: 327. 1867321 PMC1886068

[91] Hunt JS . Cytokine networks in the uteroplacental unit: macrophages as pivotal regulatory cells. J Reprod Immunol. 1989; 16: 1–17. 2689644 10.1016/0165-0378(89)90002-8

[92] Yoshinaga K . Two concepts on the immunological aspect of blastocyst implantation. J Reprod Dev. 2012; 58: 196–203. 22738903 10.1262/jrd.2011-027

[93] Bourdiec A , Martel V , Akoum A . Synchronous regulation of the determinants of endometrial receptivity to interleukin 1 at key stages of early embryo implantation *in vivo* . Fertil Steril. 2014; 101: 1183–93. 24534280 10.1016/j.fertnstert.2014.01.011

[94] D'Amora P , Sato H , Girão MJ , *et al* Polymorphisms in exons 1B and 1C of the type I interleukin‐1 receptor gene in patients with endometriosis. Am J Reprod Immunol. 2006; 56: 178–84. 16911713 10.1111/j.1600-0897.2006.00415.x

[95] Hanna N , Hanna I , Hleb M , *et al* Gestational age‐dependent expression of IL‐10 and its receptor in human placental tissues and isolated cytotrophoblasts. J Immunol. 2000; 164: 5721–8. 10820249 10.4049/jimmunol.164.11.5721

[96] Murphy SP , Fast LD , Hanna NN , *et al* Uterine NK cells mediate inflammation‐induced fetal demise in IL‐10‐null mice. J Immunol. 2005; 175: 4084–90. 16148158 10.4049/jimmunol.175.6.4084

[97] Tinsley JH , South S , Chiasson VL , *et al* Interleukin‐10 reduces inflammation, endothelial dysfunction, and blood pressure in hypertensive pregnant rats. Am J Physiol Regul Integr Comp Physiol. 2010; 298: R713–9. 20053959 10.1152/ajpregu.00712.2009

